# Utilisation of community care services and self-rated health among elderly population in China: a survey-based analysis with propensity score matching method

**DOI:** 10.1186/s12889-021-11989-x

**Published:** 2021-10-25

**Authors:** Liu Yang, Lijian Wang, Xiaodong Di, Xiuliang Dai

**Affiliations:** School of Public Policy and Administration, Xi’an Jiaotong University, People’s Republic of China, No 28 West Road, Xi’an, Xianning, 710049 Shaanxi China

**Keywords:** Community care services utilisation, Elderly health, Propensity score matching, China

## Abstract

**Background:**

Elderly care and elderly health are the enormous challenges in such an aging society as China. Community care services have been developing rapidly in recent years in China as an increasingly mainstream care resource to promote elderly health. The purpose of this study is to examine the association between using community care services and self-rated health among Chinese elderly.

**Methods:**

A cross-sectional survey was conducted in 2019 and 612 elderly people from China’s Shaanxi province were enrolled. The binary logistic regression was first employed to explore the association between community care services utilisation and elderly health. Given the potential selection bias issue, the propensity score matching method was hired to generate comparable samples between participants who used these services and participants who didn’t, and further examine the health benefits of using four types of services.

**Results:**

The results of the binary logistic regression showed that the use of community care services predicted a better health status of elderly individuals. Overall, the results of the propensity score matching method showed the similar results. Specifically, with the nearest neighbors matching algorithm, using daily care services was significantly associated with a 0.246 increase in the self-rated health of the elderly (T = 1.83). For medical care services, the mean of self-rated health of elderly individuals who used these services was 3.542, significantly higher than those who didn’t (T = 2.15). For spiritual comfort services, elderly individuals using these services showed a significant increase by 0.280 in the self-rated health (T = 1.82). For social and recreational services, the result of the nearest neighbor matching method was not statistically significant, while the results of kernel matching method and the mahalanobis matching method showed a significant increase in the self-rated health among elderly individuals using these services (T = 2.03, T = 2.03, respectively). All the estimated results passed the Rosenbaum bounds analysis and were not sensitive to hidden bias.

**Conclusions:**

Using community care services improved the self-rated health of the elderly. More effective measures may be implemented to increase access to care resources for senior citizens, and further improve their health status.

**Supplementary Information:**

The online version contains supplementary material available at 10.1186/s12889-021-11989-x.

## Background

Elderly care and elderly health are increasing concerns for countries all over the world, and China is no exception. By the end of 2020, China’ elderly population aged 65 years and over had reached 190.64 million, accounting for 13.50% of the country’s total population [1]. However, as the country with the largest elderly population in the world, the health condition of the elderly in China is not optimistic. According to the report published by the China Research Center on Aging in 2018, only a third of elderly population in China reported their health status as “good” and a high proportion of the elderly were suffering from one or more chronic diseases [2]. Therefore, effective policies and measures are in sore need to solve these crucial and enormous challenges to the Chinese elderly care and elderly health system.

In China, the care resources for elderly people are mainly provided by the family, elderly care institution and the community [3]. However, with decline of filial piety culture and the acceleration of modern industrialization in China, the role of family support is decreasing, resulting in the decline of elderly health and the increase of elderly suicide [4–6]. Additionally, due to the economic factors and living preference, the institution endowment resources only solve the care demands of a small minority of elderly population in China [7]. Hence, the critical need for community care services in China is painfully obvious and urgent.

Community care services, defined as professional care provided for the elderly living in the home and community with formally assessed demands, include in-system and out-system care services in China [4]. The Chinese government has put great emphasis on the development of community care services to respond to the rapid growth of the aging population and improve their living environments and health condition [8]. For instance, in ‘the 13^th^ five year national plan (2016-2020) for developing the elderly care system’ issued in 2017, it is required to give priority to the development of community-based care services [9].

While community care services have become an increasingly significant mode of care provision in China, few studies have examined their effects on elderly individuals’ health outcomes [10]. Some research conducted in western countries have reported the positive effect of using community-based services on the health and quality of life of older adults [11–15]. Theoretically, using community care services could maintain older adults’ social networks, thus contributing positively to their health [16]. Nevertheless, some recent literature revealed that using community care services is only effective on promoting health among seniors with consistently low daily functions or moderate loneliness and services of low quality could even exacerbate depression and medical symptoms among the elderly [17–19].

A few literatures have focused on the potential effect of community care services on the elderly health in China and provided important experimental evidences [20–23]. However, previous studies only paid attention on the perceived availability of community care services, and the effect of using these services on the elderly health is virtually unknown. Most studies only focused on the association between community care services and the mental health of elderly individuals. Moreover, all these studies hired regression analysis to examine the effect of community care services on elderly health, while the potential selection bias issue and unobservable missing variables might bias these estimations.

Therefore, to fill these research gaps, the current study assessed the utilisation of community care services and the self-rated health (SRH) of elderly people in China, and examined their relationship with the propensity score matching (PSM) method. The findings of this study could shed light on the future policy implementation on the development of community care services in China.

## Method

All methods were carried out in accordance with relevant guidelines and regulations. Study protocols and consent forms were approved by the medical ethics committee of Health Science Center of Xi’an Jiaotong University (approval number 2016–416). All participants provided informed consent to participate in the study.

### Data and sampling

The data used in this study were obtained through the survey used in the High-Quality Development of China’s Undertakings for The Aged organized by Xi’an Jiaotong University in 2019 [23]. A stratified sampling method was used to in this survey. First, three cities, namely, Baoji, Yan’an, and Hanzhong were selected, as they are representative cities of Guanzhong, Shanbei and Shannan regions of China’s Shaanxi province and pilot cities of community care services. Also, the three cities are typical aging cities and middle-ranking economic cities in China. Second, among the three cities, based on the per capita gross value of industrial output, we used the isometric random sampling method to select seven counties (districts). Third, from each county (district), we selected three or four typical communities and each community provided care services for the local older adults. Finally, according to the roster of the residents by age and the total elderly population of each selected site provided by the local residential committee, we randomly selected respondents among the elderly individuals in each community if they (1) were aged 60 years or older, (2) were able to communicate independently or communicate with the help of investigators (the elderly with severe hearing loss, obvious language difficulties, severe cognitive impairment or other communication disorders were excluded), and (3) were willing to take part in the survey.

The team members included six specialists and 21 well-trained students. The information collected for this study included four parts: (1) Personal sociodemographic characteristics; (2) community care services utilisation; (3) SRH. All questionnaires were completed anonymously and ethical guidelines were strictly followed. Data collection took approximately 20 min for each respondent. The research team had conducted a pre-survey and estimated the lowest limit of sample size based on the proportion of older adults using community care services, which is about 400. In this survey, about 700 older adults living in the community were contacted and a total of 681 elderly people agreed to participate in the survey. After filtering questionnaires that amounts of data about personal sociodemographic characteristics, community care services utilisation and SRH were missing, 612 valid samples were obtained and the final response rate of this survey was 89.87%.

### Measurement

#### Outcome variables

SRH was used as an outcome variable to measure an elderly individual’s health status in this study. SRH is a common indicator that can comprehensively reflect individuals’ self-perception of their health and a good predictor of objective health outcomes, including morbidity and mortality [24–27], which has been hired to proxy health status in numerous studies [28, 29]. Hence, we assessed the SRH using the question “In general, how would you rate your health status?” with five possible answer categories: 5 (“Very good”), 4 (“Good”), 3 (“Fair”), 2 (“Bad”), and 1 (“Very bad”). The higher score indicates a higher level of an elderly individual’s health status. In addition, the binary indicator for SRH in which healthy = 1 (based on responses of very good and good) and unhealthy = 0 (based on responses of fair, bad and very bad) was further constructed.

#### Treatment variables

Community care services for the elderly are diverse in China. This study listed 22 specific community care services and respondents were asked whether they had used each of them [30]. Some living support services, such as housekeeping, grocery delivery and community canteen services, were grouped into an aggregated treatment category “daily care services utilisation”. Likewise, different health-related services, such as health lectures, regular medical examinations and visiting medical services, were aggregated into the treatment category “medical care services utilisation”. For the treatment categories “social and recreational services utilisation”, some entertainment and cultural activities and services, such as joining interest groups, recreation centers and chess and card clubs were included. Similarly, the services related to elderly people’s spiritual needs, like psychological counselling and matrimonial services were grouped into the treatment category “spiritual comfort services utilisation”. Once a respondent said that he/she used one or more specific services, the corresponding treatment category was defined as 1. If a respondent didn’t use any service, the corresponding category was defined as 0.

#### Covariates

We used 16 covariates to model the propensity of the elderly to be in different health status and adjust the models. The covariates included demographic variables, such as age, gender, marital status, education level, chronic diseases and activities of daily living (ADL) limitations, and socioeconomic variables, such as hukou location, household income, health insurance and old-age insurance. In addition, considering the potential effect of family support on community care services utilisation and the health of older adults, we used a series of family variables as covariates, including number of children, instrumental support (measured by asking respondents whether they got sufficient economic support and daily care from family members) and emotional support (measured by asking respondents whether they communicated frequently with family members). Rubin advised that as many covariates as possible should be included in the model predicting propensity score to receive treatment, in order to maximally reduce the potential for selection bias – even those that only weakly predict the treatment [31]. Therefore, we also included other covariates that may affect community care services utilisation and the SRH of older adults, including regular exercise, outpatient service utilisation and inpatient service utilisation. In this study, outpatient visits in the last two weeks and inpatient visits in the past year were selected as the indicators to measure the outpatient service utilisation and the inpatient service utilisation respectively. A description of these covariates is shown in Table 1.

**Table 1:** Definition/codes of the covariates

Variable	Codes/definition
Age	Continuous variable
Gender	Binary variable (1 = Male; 0 = Female)
Marital status ^a^	Binary variable (1 = Non-single; 0 = Single)
Education level	Categorical variable (primary school or lower; junior middle school; senior middle school or higher)
ADL limitation ^b^	Binary variable (1 = Yes; 0 = No)
Chronic disease	Binary variable (1 = Yes; 0 = No)
Hukou location	Binary variable (1 = Urban; 0 = Rural)
Annual income ^c^	Binary variable (1 = Above-average incomes; 0 = Below-average incomes)
Health insurance	Binary variable (1 = Yes; 0 = No)
Old-age insurance	Binary variable (1 = Yes; 0 = No)
Outpatient service	Binary variable (1 = Yes; 0 = No)
Inpatient service	Binary variable (1 = Yes; 0 = No)
Number of children	Continuous variable
Instrumental support	Binary variable (1 = Yes; 0 = No)
Emotional support	Binary variable (1 = Yes; 0 = No)
Regular exercise	Binary variable (1 = Yes; 0 = No)

a. Non-single refers to the state of being married, Single contains unmarried, divorced and widowed;

b. ADL limitation was measured by asking respondents whether they had difficulty or needed assistance in performing any of the following ADLs: feeding, bathing, dressing, going to the toilet, continence, indoor mobility and walking outdoors. The respondents were classified as ‘Yes’ if they needed assistance with at least one activity.

c. Annual income contains a participant’s pensions, economic support from children, incomes from migrant working or farming and other incomes. Average incomes: the mean value of the total annual household incomes of 612 respondents.

### Statistical method

All the analyses were conducted in STATA version 15.1. First, the descriptive statistics were conducted to show the characteristics of the covariates and the treatment variables. Second, the binary logistic regression analysis was used to investigate the relationship between SRH, community care services utilisation and covariates. Meanwhile, we used the multivariate linear regression analysis to test the robustness of the binary logistic regression analysis results. Third, considering the potential selection bias issue in community care services utilisation and health studies, the PSM method was further hired to examine the effect of community care services utilisation on elderly individuals’ health status.

PSM is a two-stage process. In the first stage, a logistic regression model was used to calculate all respondents’ propensity scores for using community care services based on the 16 covariates mentioned above. The propensity score is defined as follows:

1 $$ logit(P)=\log \left(\frac{1}{1-P}\right)=\mu +\beta x $$

where *P* is the probability that the individual respondent uses community care services; *x* is a vector of characteristics correlated with elderly individuals’ community care services utilisation and SRH; *β* is a vector of parameters to be estimated; and *μ* is the intercept term corresponding to community care services utilisation.

In the second stage, the estimated propensity scores obtained in the first stage were used to match the elderly sample. In this stage, it is necessary to check the covariate balancing (matching quality) between elderly individuals that use and do not use community care services. The two-sample t-test was used to assess the matching quality and if there are no significant differences of covariates between the elderly groups that used and did not use community care services after matching, the matching result is seen as a success [32]. Also, we discussed the matching quality by examining the degree to which the estimated propensity scores for the treatment group (use community care services) and control group (do not use community care services) overlap with histograms. Based on that, three matching algorithms, including the nearest neighbors matching algorithm, the kernel matching algorithm and the mahalanobis algorithm, were used to estimate the average treatment effect on the treated (ATT) of four types of community care services utilisation on elderly health.

Finally, Rosenbaum bounds method was used to assess the degree of the hidden bias and check the sensitivity of results. According to the theory of Rosenbaum bounds method, the critical level of hidden bias gamma, which is the log odds of differential assignment due to unobserved characteristics, is the value when the upper bound significance level (sig +) is approaching 5%. The higher the value of gamma the less possibility that the results are sensitive to hidden bias [33]. There is debate on selecting the threshold of gamma value to examine the existence of hidden bias. Based on previous studies, we set 1.15 as the threshold to analyze the sensitivity [33–35]. It is worth noting that the bounds analysis could advise some caution when interpreting the results, but it does not imply existence of unobservable [36].

## Results

### Descriptive analysis

Table 2 summarizes participants’ characteristics. Among these 612 participants, the average age of these older adults was 70.22 years, with a range of 60 to 92 years. 38.24% of these participants were male and 72.55% of them were non-single. 43.79% of participants had a low educational level. Only 11 participants reported that they have at least one ADL limitation, while 350 participants suffered from chronic diseases. 344 participants were urban residents and 301 participants had a household income above the average level. Most of the elderly individuals had the health insurance (98.20%) and old-age insurance (96.41%). 95 participants visited outpatient in the last two weeks and 83 participants visited inpatient in the past year. The average number of children of these participants was 2.43. 524 respondents reported receiving sufficient economic support and daily care from family members, and 525 communicated frequently with family members. 486 participants had regular exercise.

**Table 2:** The descriptive statistics of covariates

Variable		Number	Percentage
Age	(mean, SD)	70.22 (7.07)	–
Sex	Male	234	38.24%
	Female	378	61.76%
Marital status	Non-single	444	72.55%
	Single	168	27.45%
Education level	Primary school and lower	268	43.79%
	Middle school	177	28.92%
	Senior middle school and higher	167	27.29%
ADL limitation	Yes	11	1.80%
	No	601	98.20%
Chronic disease	Yes	350	57.19%
	No	262	42.81%
Hukou location	Urban	344	56.21%
	Rural	268	43.79%
Household income	Above-average incomes	301	49.18%
	Below-average incomes	311	50.82%
Health insurance	Yes	601	98.20%
	No	11	1.80%
Old-age insurance	Yes	590	96.41%
	No	22	3.59%
Outpatient service	Yes	95	15.52%
	No	517	84.48%
Inpatient service	Yes	83	13.56%
	No	529	86.44%
Number of children	(mean, SD)	2.43 (1.25)	–
Instrumental support	Yes	524	85.62%
	No	88	14.38%
Emotional support	Yes	525	85.78%
	No	87	14.22%
Regular exercise	Yes	486	79.41%
	No	126	20.59%

Table 3 presents the community care services utilisation of the participants and the mean value and standard error of SRH scores of participants who used and didn’t use community care services. Among 612 participants, compared with participants who didn’t use any community care services, the elderly using these services reported a higher level of SRH.

**Table 3:** The descriptive statistics of treatment variables

Treatment Variables		Number	Percentage	SRH	
				Mean	SE
Daily care services	Yes	160	26.14%	3.68	1.00
	No	452	73.86%	3.39	1.04
Medical care services	Yes	462	75.49%	3.55	1.00
	No	150	24.51%	3.23	1.09
Social and recreational services	Yes	388	63.40%	3.61	0.97
	No	224	36.60%	3.21	1.09
Spiritual comfort services	Yes	91	14.87%	3.71	0.92
	No	521	85.13%	3.42	1.05

SE: standard error.

### Binary logistic regression analysis

Table 4 presents the empirical results derived via the binary logistic regression models. All estimate results of the effects of four types of community care services utilisation on elderly individuals’ health status incurred significant and positive coefficients. Specifically, elderly individuals’ using daily care services reported a better SRH than those who didn’t use any daily care service (β = 0.735, *p* = 0.213 in Model 1). After controlling for covariates, using medical care services were significantly and positively associated with SRH among the elderly (β = 0.768, *p* = 0.216 in Model 2). Similarly, participants using social and recreational services and spiritual comfort services were more likely to report a better SRH than those who didn’t (β = 0.442, *p* = 0.197 in Model 3; β = 0.758, *p* = 0.269 in Model 4).

**Table 4:** Association between SRH and community care service (Binary logistic regression)

Variables	Model 1	Model 2	Model 3	Model 4
	β (SE)	β (SE)	β (SE)	β (SE)
Daily care services (ref: no)	0.735 (0.213) ***			
Medical care services (ref: no)		0.768(0.216) ***		
Social and recreational services (ref: no)			0.442 (0.197) **	
Spiritual comfort services (ref: no)				0.758 (0.269) ***
Age	−0.058 (0.015) ***	− 0.059(0.015) ***	− 0.055(0.015) ***	− 0.054(0.015) ***
Gender (ref: Female)	− 0.113 (0.196)	− 0.133(0.197)	−0.119(0.195)	− 0.144(0.197)
Marital status (ref: single)	0.075 (0.222)	0.040(0.220)	0.015(0.219)	0.099(0.223)
Education level 2(ref: Education level 1)	0.066(0.235)	0.107(0.235)	0.106(0.233)	0.114(0.234)
Education level 3(ref: Education level 1)	0.230(0.261)	0.285 (0.259)	0.304 (0.257)	0.343(0.258)
ADL limitation(ref: no)	−1.398 (0.876)	−1.469(0.862) *	− 1.202(0.856)	−1.407(0.857)
Chronic disease(ref: no)	−0.915 (0.185) ***	− 0.857(0.185) ***	− 0.862(0.184) ***	− 0.853(0.184) ***
Hukou location(ref: rural)	0.457 (0.198) **	0.482(0.199) **	0.480(0.199) **	0.390(0.198) **
Household income(ref: below-average incomes)	0.709 (0.188) ***	0.770(0.189) ***	0.709(0.187) ***	0.785(0.190) ***
Health insurance(ref: no)	0.794 (0.760)	0.459 (0.763)	0.662(0.749)	0.753(0.753)
Old-age insurance(ref: no)	0.599 (0.541)	0.686 (0.541)	0.533(0.536)	0.678(0.557)
Outpatient service(ref: no)	−0.658 (0.259) **	−0.498(0.260) *	− 0.668(0.259) **	− 0.628(0.259) **
Inpatient service(ref: no)	− 0.517 (0.276) *	− 0.470(0.277) *	− 0.360(0.277)	− 0.481(0.274) *
Number of Children	0.103 (0.084)	0.094 (0.085)	0.146(0.085) *	0.116(0.084)
Instrumental support(ref: no)	−0.335 (0.324)	−0.300(0.323)	− 0.395(0.325)	−0.463(0.327)
Emotional support(ref: no)	0.379(0.317)	0.361(0.319)	0.411(0.318)	0.455(0.317)
Regular exercise(ref: no)	0.348(0.231)	0.442(0.232) *	0.413(0.229) *	0.366(0.231)
Observation	612	612	612	612
Adjusted R-squared	0.140	0.141	0.132	0.136

β: regression coefficient; SE: standard error.

Education level 1: primary school or lower; Education level 2: junior middle school; Education level 3: senior middle school or higher.

* *p* < 0.1, ** *p* < 0.05, and *** *p* < 0.01

To ensure the reliability of the binary logistic regression analysis results in Table 4, we further changed the testing model to test the robustness of the results. According to the results of multivariate linear regression analysis (see Supplementary Table 1, Additional File 1), the significance and direction of the coefficient of dependent variables (four types of community care services) in Model 5 to Model 8 were consistent with that in Model 1 to Model 4, suggesting the robust and reliable results.

### Propensity score matching estimates

First, we present the results of the logistic regression models (Model 9 to Model 12) used to calculate all respondents’ propensity scores for using four types community care services based on the 16 covariates in Table 5. Our results showed that the use of four types of community care services was significantly influenced by different covariates.

**Table 5:** Estimates of respondents’ propensity scores for using four types of community care services

Covariate	Daily care services	Medical care services	Social and recreational services	Spiritual comfort services
	Model 9	Model 10	Model 11	Model 12
	β (SE)	β (SE)	β (SE)	β (SE)
Age	0.003 (0.016)	0.002 (0.016)	−0.028 (0.015) *	−0.041 (0.020) **
Gender (ref: Female)	0.042 (0.208)	0.252 (0.219)	0.193 (0.202)	0.390 (0.260)
Marital status (ref: single)	−0.543 (0.227) **	−0.264 (0.243)	− 0.173 (0.222)	− 1.032 (0.277) ***
Education level 2(ref: Education level 1)	0.410 (0.260)	0.167 (0.253)	0.176 (0.234)	0.196 (0.318)
Education level 3(ref: Education level 1)	0.954 (0.276) ***	0.641 (0.288) **	0.608 (0.267) **	0.240 (0.338)
ADL limitation(ref: no)	0.333 (0.729)	0.258 (0.811)	−1.443 (0.755) *	0.733 (0.933)
Chronic disease(ref: no)	0.204 (0.198)	−0.230 (0.204)	− 0.126 (0.188)	− 0.30 (0.243)
Hukou location(ref: rural)	− 0.159 (0.214)	− 0.417 (0.219) *	− 0.626 (0.206) ***	0.405 (0.266)
Household income (ref: below-average incomes)	0.065 (0.200)	−0.339 (0.208)	0.072 (0.192)	−0.674 (0.254) ***
Health insurance(ref: no)	0.252 (0.819)	2.382 (0.765) ***	1.585 (0.748) **	0.490 (1.152)
Old-age insurance(ref: no)	−0.258 (0.527)	−1.085 (0.662)	0.207 (0.493)	−0.682 (0.582)
Outpatient service(ref: no)	0.250 (0.268)	−0.784 (0.259) ***	0.541 (0.268) **	−0.075 (0.362)
Inpatient service(ref: no)	0.326 (0.281)	−0.128 (0.285)	−1.070 (0.268) ***	0.283 (0.355)
Number of children	0.089 (0.088)	0.192 (0.094) **	−0.298 (0.084) ***	−0.003 (0.114)
Instrumental support(ref: no)	−0.320 (0.352)	− 0.458 (0.348)	0.291 (0.319)	1.325 (0.553) **
Emotional support(ref: no)	0.620 (0.372) *	0.659 (0.326) **	0.422 (0.311)	−0.108 (0.471)
Regular exercise(ref: no)	0.791 (0.275) ***	−0.022 (0.252)	0.246 (0.231)	0.883 (0.369) **
Observation	612	612	612	612
Pseudo R-squared	0.049	0.064	0.114	0.089

β: regression coefficient; SE: standard error.

Education level 1: primary school or lower, Education level 2: junior middle school, Education level 3: senior middle school or higher.

* *p* < 0.1, ** p < 0.05, and *** *p* < 0.01

Second, the two-sample t-test were conducted. After matching, all the *P* values were larger than 0.05, indicating the satisfied balancing property and good matching performance (see Supplementary Table 2, Additional File 1). In addition, we plotted the histograms of propensity scores for the treatment and control groups, which showed sufficient overlap between these two groups and thus supported the overlap or common support assumption for PSM (see Supplementary Fig. 1 to Supplementary Fig. 4, Additional File 1).

Finally, we estimated the ATT of four types of community care services utilisation on the SRH of the elderly with three matching algorithms and the results are present in Table 6. To be specific, the result of the nearest neighbor matching method showed that using daily care services was significantly associated with a 0.246 increase in the average SRH scores of the elderly (T = 1.83, *p* < 0.1). The mean of SRH scores of elderly individuals who did not use any medical care service was 3.221, while the mean of elderly individuals using medical care services was 3.542, reporting a significant increase (T = 2.15, *p* < 0.05). Similarly, for spiritual comfort services, the estimated ATT from the nearest neighbor matching method increased by 0.280 and was statistically significant (T = 1.82, *p* < 0.1). With the nearest neighbor matching method, the effect of using social and recreational services on the SRH of the elderly was not statistically significant (T = 1.43, *p* > 0.1). However, the results of kernel matching method and the mahalanobis matching method showed a significant increase in the average SRH scores of elderly individuals who used this type of services compared with those who did not (T = 2.03, T = 2.37, *p* < 0.05 respectively). Combining the result of binary logistic regression method, it is believed that social and recreational services utilisation was significantly associated with a better health status of older adults and our results were generally robust.

**Table 6:** Estimated ATT of four types of community care services on the SRH of participants

Treatment Variables	Matching algorithm	Treated	Control	Difference	SE	T
Daily care services	NN	3.675	3.429	0.246	0.134	1.83 *
	Kernel	3.675	3.436	0.239	0.097	2.47 **
	Mahalanobis	3.675	3.456	0.219	0.093	2.35 **
Medical care services	NN	3.542	3.221	0.321	0.149	2.15 **
	Kernel	3.542	3.302	0.240	0.109	2.20 **
	Mahalanobis	3.545	3.366	0.179	0.112	1.60
Social and recreational services	NN	3.616	3.422	0.194	0.135	1.43
	Kernel	3.616	3.405	0.211	0.104	2.03 **
	Mahalanobis	3.613	3.383	0.230	0.097	2.37 **
Spiritual comfort services	NN	3.733	3.454	0.280	0.154	1.82 *
	Kernel	3.733	3.510	0.223	0.114	1.96 **
	Mahalanobis	3.714	3.489	0.225	0.113	2.00 **

SE: standard error.

NN: nearest - neighbour matching.

* p < 0.1, ** p < 0.05, and *** p < 0.01

### Sensitivity analysis

Rosenbaum boundary estimation results are shown in Supplementary Table 3 (see Supplementary Table 3, Additional File 1). All the critical level of hidden bias gamma of these results were higher than 1.15. Meanwhile, given the fact that a large number of covariates were controlled in this study, it is reasonable to believe that these results are not sensitive to hidden bias.

## Discussion

To our knowledge, it is the first study to examine the association between using community care services and elderly health with the PSM method in China. In the treatment effect evaluation study with observational data, comparisons between treatment and control are hampered by the high dimensionality of the observed characteristics [37]. PSM does well to yield unbiased estimates of the treatment impact by controlling for all the confounding effects of a large number of covariates and the elderly’ attitude towards community care services utilisation in order to infer causality [38–40]. Overall, the results of the PSM method were consistent with that of the binary logistic regression method, suggesting significant and positive association between using community care services and SRH among the studied participants. However, the discrepancy of the size of the effects between two estimation method indicated signs of selection bias issue and we mainly discussed the results derived via the PSM method.

For daily care services, the results of three matching algorithms showed that using daily care services positively and significantly affects the elderly health. Existing findings have proved that the use of home-delivered meal services or community canteen services, which are typical forms of community daily care services, has significantly positive impact on the nutritional intake of community living older adults, thus improving their health status [41–43]. Moreover, another possible explanation for our finding is that daily care services provided by the community may liberate older adults from burdensome housework and allow them to spare more time for exercise and relaxation, which might be benefit for their physical health and mental health [10, 20].

For medical care services, while the result derived via the mahalanobis matching algorithm did not pass the significance test at the 10% level, the results of other two algorithms indicated a significant increase of SRH scores among elderly people who used medical care services. Overall, we believed that there is a significant and positive association between medical care services utilisation and elderly health. This finding is in line with the previous studies which found that the community health promotion programs could help older adults increase health knowledge and their awareness of health management and thus may be effective in preventing the onset of disability, chronic diseases and depressive symptoms [44–46]. In China, with the health care reforms and the construction of three-tiered healthcare system, more efforts have been made to establish community healthcare centers and improve their capacity and quality of disease prevention and health promotion [47, 48], attracting more Chinese seniors to use the community care services and thus improving their health status.

For social and recreational services, results of two algorithms passed the significance test at the 5% level and suggested the positive and significant association between social and recreational services utilisation and elderly health, which is consistent with previous research [49]. A typical form of community social and recreational services in China is providing leisure activities appropriate to older adults in some elderly activity centers, which has been proved to be an effective intervention for the improvement of health and life satisfaction of the elderly [50, 51]. In addition, community social and recreational services provide the elderly with a direct social engagement way to obtain social resources and social capital. At present, it is a global consensus that social engagement has a positive impact on the physical and mental health of elderly individuals through communication and social support [52]. Therefore, this might help to explain the health benefits of using social and recreational services in this study.

For spiritual comfort services, our findings suggested a significantly positive association between spiritual comfort services utilisation and elderly health. There is an increasing concern on the psychological health problems of the elderly and the spirit and psychological services in the community are in high demand [53]. Traditionally, the family is the main source of spiritual and emotional support for the Chinese elderly [54]. However, as the traditional intergenerational co-residence mode has been decreasing and the traditional perception has been eroding in the past two decades, community care services have been emerging as a supplement of family support [55]. Particularly, spiritual comfort services, like psychological counselling and company of volunteers, provides older adults with professional psychological services and works as a new way to help them regain emotional and social support, which may make them feel supported and secure, and further improve their self-perceived health [8].

This study seeks to strengthen our understanding of the health benefits of using different types of community care services and presents important policy implications. To improve the health status of elderly individuals living in the home and the community, more financial support and human resources are expected to be put to continue expanding community care services coverage and improve the accessibility of these services for the elderly in China, especially in rural areas and economically undeveloped area. Furthermore, not only should the policy-makers make efforts to achieve further progress in the quantity of community care services, but they need to pay more attention on the high-quality development of these services, in particular medical care services and spiritual comfort services, which ask for high professional level, thus attracting more older adults to get involved in community care services.

It is important to bear in mind that there are some limitations in this study. First, for the PSM estimated effects of using community care services in this study, while we have controlled for a large number of covariates and conducted the sensitivity check of our results, it is impossible to completely solve the selection bias issue. Second, in the process of the PSM method, it is inevitable to drop some incomparable samples and decrease sample size, which may lead to some potential bias. Third, in the sampling process, the lack of using a professional instrument to measure the elderly’s cognition may lead to some social desirability bias in this study. Finally, considering the special separate urban-rural structure and regional economic disparity of China, the effect of using community care services may vary in the specific rural and urban contexts, hence the heterogeneity effect of rural and urban contexts may be included in further studies.

## Conclusion

Applying the PSM method to partly address potential selection bias issues, the current study found that using daily care services, medical care services, social and recreational services and spiritual comfort services had an overall significant and positive effect on SRH of the Chinese elderly. The findings of this study provide a better understanding about the association between community care services utilisation and health in China and imply that policy-makers should make more efforts on expanding community care services coverage and promoting the quality of these services to increase access to care resources for elderly individuals, thus improving their health status.

## Supplementary Information

**Additional file 1.**

## Data Availability

The datasets used and/or analyzed in this study belong to our research team, and data does not involve any personal privacy information. The datasets are available from the corresponding author on reasonable request.

## Abbreviations

SRH: self-rated health
PSM: propensity score matching
ADL: activities of daily living
ATT: the average treatment effect on the treated
