# Supplementary material for: Utilisation of community care services and self-rated health among elderly population in China: a survey-based analysis with propensity score matching method
Source: BMC Public Health. 2021 Oct 25;21:1936. doi: 10.1186/s12889-021-11989-x (PMC8546940; doi:10.1186/s12889-021-11989-x)
Supplement: Supplementary file 1 — Additional file 1. [file 12889_2021_11989_MOESM1_ESM.docx]

**Supplementary Materials**

**Supplementary Table 1** Robustness test: multivariable regression results of the linear regression models

| **Variables** | **Model 5** | **Model 6** | **Model 7** | **Model 8** |
| --- | --- | --- | --- | --- |
|  | **β (SE)** | **β (SE)** | **β (SE)** | **β (SE)** |
| Daily care services  (ref: no) | 0.264  (0.087) *** |  |  |  |
| Medical care services  (ref: no) |  | 0.270  (0.089) *** |  |  |
| Social and recreational services (ref: no) |  |  | 0.213  (0.083) ** |  |
| Spiritual comfort services  (ref: no) |  |  |  | 0.242  (0.108) ** |
| Age | -0.026  (0.006) *** | -0.0264  (0.006) *** | -0.025 (0.006) *** | -0.025 (0.006) *** |
| Gender  (ref: Female) | -0.060  (0.082) | -0.070  (0.082) | -0.066 (0.082) | -0.069 (0.082) |
| Marital status  (ref: single) | 0.080  (0.091) | 0.065 (0.091) | 0.060  (0.091) | 0.083 (0.092) |
| Education level 2 (ref: Education level 1) | 0.110  (0.098) | 0.122 (0.098) | 0.122 (0.098) | 0.123 (0.098) |
| Education level 3 (ref: Education level 1) | 0.267  (0.109) ** | 0.285  (0.108) *** | 0.289  (0.108) *** | 0.309 (0.108) *** |
| ADL limitation (ref: no) | -0.827  (0.284) *** | -0.824 (0.284) *** | -0.750 (0.286) *** | -0.834 (0.285) *** |
| Chronic disease (ref: no) | -0.485  (0.077) *** | -0.464 (0.077) *** | -0.470 (0.077) *** | -0.467 (0.077) *** |
| Hukou location (ref: rural) | 0.074  (0.082) | 0.086 (0.082) | 0.094 (0.083) | 0.055 (0.083) |
| Household income (ref: below-average incomes) | 0.376  (0.078) *** | 0.395  (0.078) | 0.376 (0.078) | 0.399 (0.079) |
| Health insurance (ref: no) | 0.549  (0.288) * | 0.429  (0.291) | 0.491 (0.290) * | 0.551 (0.289) * |
| Old-age insurance (ref: no) | 0.157  (0.207) | 0.189  (0.207) | 0.135 (0.207) | 0.164 (0.208) |
| Outpatient service (ref: no) | -0.131  (0.106) | -0.077  (0.107) | -0.142 (0.107) | -0.116 (0.107) |
| Inpatient service (ref: no) | -0.347  (0.112) *** | -0.325  (0.112) *** | -0.281 (0.114) *** | -0.337 (0.113) *** |
| Number of Children | 0.042  (0.035) | 0.0384  (0.035) | 0.061 (0.035) * | 0.047 (0.035) |
| Instrumental support (ref: no) | -0.066  (0.133) | -0.056 (0.133) | -0.092 (0.133) | -0.108 (0.134) |
| Emotional support (ref: no) | 0.153  (0.131) | 0.146 (0.131) | 0.159 (0.131) | 0.180 (0.131) |
| Regular exercise (ref: no) | 0.157  (0.095) * | 0.192 (0.094) ** | 0.181 (0.095) * | 0.171 (0.095) * |
| Observation | 612 | 612 | 612 | 612 |
| Adjusted R-squared | 0.2378 | 0.238 | 0.234 | 0.232 |

β: regression coefficient; SE: standard error.

Education level 1: primary school or lower; Education level 2: junior middle school; Education level 3: senior middle school or higher.

* p < 0.1, ** p < 0.05, and *** p < 0.01.

**Supplementary Table 2** Balancing test for covariates before and after matching

| **Covariate** | **Unmatched** | **P value** | | | |
| --- | --- | --- | --- | --- | --- |
|  | **Matched** | **Daily care**  **services** | **Medical care**  **services** | **Social and**  **recreational services** | **Spiritual comfort services** |
| Age | U | 0.417 | 0.697 | 0.000 | 0.068 |
|  | M | 0.244 | 0.844 | 0.268 | 0.539 |
| Gender | U | 0.730 | 0.071 | 0.044 | 0.454 |
|  | M | 0.731 | 0.590 | 0.662 | 0.880 |
| Marital status | U | 0.061 | 0.504 | 0.158 | 0.011 |
|  | M | 0.558 | 0.662 | 0.624 | 0.878 |
| Education level 2 | U | 0.796 | 0.588 | 0.484 | 0.502 |
|  | M | 0.803 | 0.563 | 0.107 | 0.277 |
| Education level 3 | U | 0.002 | 0.144 | 0.000 | 0.288 |
|  | M | 0.493 | 0.715 | 0.347 | 0.630 |
| ADL limitation | U | 0.932 | 0.623 | 0.012 | 0.756 |
|  | M | 0.475 | 0.817 | 0.654 | 0.563 |
| Chronic disease | U | 0.228 | 0.323 | 0.181 | 0.106 |
|  | M | 0.176 | 0.099 | 1.000 | 0.657 |
| Hukou location | U | 0.348 | 0.206 | 0.125 | 0.043 |
|  | M | 0.733 | 0.353 | 0.428 | 0.757 |
| Household income | U | 0.429 | 0.327 | 0.716 | 0.027 |
|  | M | 0.057 | 0.236 | 0.517 | 0.880 |
| Health insurance | U | 0.545 | 0.000 | 0.012 | 0.587 |
|  | M | 0.653 | 1.000 | 0.654 | 1.000 |
| Old-age insurance | U | 0.903 | 0.483 | 0.381 | 0.292 |
|  | M | 0.778 | 0.615 | 0.557 | 0.734 |
| Outpatient service | U | 0.422 | 0.001 | 0.383 | 0.506 |
|  | M | 0.667 | 0.043 | 0.923 | 0.534 |
| Inpatient service | U | 0.376 | 0.202 | 0.000 | 0.827 |
|  | M | 0.877 | 0.921 | 0.621 | 0.663 |
| Number of Children | U | 0.900 | 0.156 | 0.000 | 0.115 |
|  | M | 0.246 | 0.003 | 0.582 | 0.620 |
| Instrumental support | U | 0.792 | 0.908 | 0.010 | 0.022 |
|  | M | 0.751 | 0.711 | 0.077 | 0.518 |
| Emotional support | U | 0.131 | 0.127 | 0.003 | 0.201 |
|  | M | 0.858 | 0.096 | 0.110 | 0.603 |
| Regular exercise | U | 0.003 | 0.795 | 0.154 | 0.014 |
|  | M | 0.742 | 0.088 | 0.285 | 0.506 |

Education level 2: junior middle school, Education level 3: senior middle school or higher.

**Supplementary Figure 1** Histogram of propensity scores (Daily care services)

**Supplementary Figure 2** Histogram of propensity scores (Medical care services)

**Supplementary Figure 3** Histogram of propensity scores (Social and recreational services)

**Supplementary Figure 4** Histogram of propensity scores (Spiritual comfort services)

**Supplementary Table 3** Sensitivity analysis for effects of community care services utilisation on elderly health

| Gamma | Daily care services | | Medical care services | | Social and recreational services | | Spiritual comfort services | |
| --- | --- | --- | --- | --- | --- | --- | --- | --- |
|  | sig+ | sig- | sig+ | sig- | sig+ | sig- | sig+ | sig- |
| 1.00 | 0.003 | 0.003 | 0.000 | 0.000 | 0.000 | 0.000 | 0.005 | 0.005 |
| 1.05 | 0.006 | 0.001 | 0.001 | 0.000 | 0.000 | 0.000 | 0.009 | 0.003 |
| 1.10 | 0.011 | 0.000 | 0.003 | 0.000 | 0.000 | 0.000 | 0.015 | 0.002 |
| 1.15 | 0.020 | 0.000 | 0.009 | 0.000 | 0.001 | 0.000 | 0.023 | 0.001 |
| 1.20 | 0.034 | 0.000 | 0.025 | 0.000 | 0.004 | 0.000 | 0.034 | 0.000 |
| 1.25 | 0.054 | 0.000 | 0.055 | 0.000 | 0.009 | 0.000 | 0.048 | 0.000 |
| 1.30 | 0.081 | 0.000 | 0.108 | 0.000 | 0.021 | 0.000 | 0.066 | 0.000 |
| 1.35 | 0.116 | 0.000 | 0.186 | 0.000 | 0.043 | 0.000 | 0.087 | 0.000 |
| 1.40 | 0.158 | 0.000 | 0.287 | 0.000 | 0.077 | 0.000 | 0.112 | 0.000 |
| 1.45 | 0.207 | 0.000 | 0.404 | 0.000 | 0.128 | 0.000 | 0.140 | 0.000 |
| 1.50 | 0.262 | 0.000 | 0.526 | 0.000 | 0.196 | 0.000 | 0.172 | 0.000 |
| 1.55 | 0.321 | 0.000 | 0.641 | 0.000 | 0.278 | 0.000 | 0.206 | 0.000 |
| 1.60 | 0.383 | 0.000 | 0.742 | 0.000 | 0.372 | 0.000 | 0.243 | 0.000 |
| 1.65 | 0.446 | 0.000 | 0.824 | 0.000 | 0.470 | 0.000 | 0.282 | 0.000 |
| 1.70 | 0.509 | 0.000 | 0.885 | 0.000 | 0.567 | 0.000 | 0.323 | 0.000 |
| 1.75 | 0.569 | 0.000 | 0.929 | 0.000 | 0.658 | 0.000 | 0.364 | 0.000 |
| 1.80 | 0.627 | 0.000 | 0.958 | 0.000 | 0.738 | 0.000 | 0.406 | 0.000 |
| 1.85 | 0.680 | 0.000 | 0.976 | 0.000 | 0.806 | 0.000 | 0.447 | 0.000 |
| 1.90 | 0.729 | 0.000 | 0.987 | 0.000 | 0.860 | 0.000 | 0.488 | 0.000 |
| 1.95 | 0.772 | 0.000 | 0.993 | 0.000 | 0.902 | 0.000 | 0.528 | 0.000 |
| 2.00 | 0.810 | 0.000 | 0.996 | 0.000 | 0.934 | 0.000 | 0.567 | 0.000 |

The results of the nearest neighbors matching algorithm were used for sensitivity analysis.

sig +: Upper bound significance level (overestimation of treatment effect).

sig –: Lower bound significance level (underestimation of treatment effect).
